# Influence of the workplace on learning physical examination skills

**DOI:** 10.1186/1472-6920-14-61

**Published:** 2014-03-28

**Authors:** Robbert Duvivier, Renée Stalmeijer, Jan van Dalen, Cees van der Vleuten, Albert Scherpbier

**Affiliations:** 1 Skillslab, Faculty of Health Medicine and Life Sciences, Maastricht University, Maastricht, The Netherlands; 2 Department of Educational Development and Research, Faculty of Health Medicine and Life Sciences, Maastricht University, Maastricht, The Netherlands; 3 Institute for Medical Education, Faculty of Health Medicine and Life Sciences, Maastricht University, Maastricht, The Netherlands

## Abstract

**Background:**

Hospital clerkships are considered crucial for acquiring competencies such as diagnostic reasoning and clinical skills. The actual learning process in the hospital remains poorly understood. This study investigates how students learn clinical skills in workplaces and factors affecting this.

**Methods:**

Six focus group sessions with 32 students in Internal Medicine rotation (4–9 students per group; sessions 80–90 minutes). Verbatim transcripts were analysed by emerging themes and coded independently by three researchers followed by constant comparison and axial coding.

**Results:**

Students report to learn the systematics of the physical examination, gain agility and become able to recognise pathological signs. The learning process combines working alongside others and working independently with increasing responsibility for patient care. Helpful behaviour includes making findings explicit through patient files or during observation, feedback by abnormal findings and taking initiative. Factors affecting the process negatively include lack of supervision, uncertainty about tasks and expectations, and social context such as hierarchy of learners and perceived learning environment.

**Conclusion:**

Although individual student experiences vary greatly between different hospitals, it seems that proactivity and participation are central drivers for learning. These results can improve the quality of existing programmes and help design new ways to learn physical examination skills.

## Background

A large part of medical education is situated in the workplace. This educational environment is however entirely different from the environment found during the pre-clinical years in medical school [1-3]. And although clerkships are considered crucial for acquiring a range of competencies, such as diagnostic reasoning and physical examination skills, the actual learning process in the clinical learning environment remains poorly understood [4]. Merely placing students in a clinical setting does not automatically lead to learning [5], and offering learning opportunities does not mean that students automatically make the most of them [6]. Several attempts have been made to understand learning processes in clinical practice but the actual components that constitute the learning experience remain underexposed [7-9].

The transition from the pre-clinical years to the clinical workplace is known to be a difficult period for students [10]. The sharp contrast experienced during this period has been named ‘shock of practice’ [11]. One of the problems reported by students is difficulty in applying previously acquired knowledge and skills to real patient problems [12,13]. However, the level of students’ prior knowledge and skills seems to have limited influence on performance during the transition period. On the longer term, the perceived difficulty of the transition period in their first clerkship has no negative effect on student progress [14]. After the initial period of ‘shock of practice’ students are able to use their knowledge and skills in real-life patient situations [15].

Researchers’ attention has mostly been focused on the contribution of clerkships to students’ knowledge [16] or their professional attitude [17,18] and socialisation into the profession [19]. Little is known however about the value of clinical placements in the training of physical examination skills. Observations made in the past decades gave rise to concerns about the effectiveness of clerkships for developing physical examination skills [20]. Undergraduate clinical training was perceived as inadequate in terms of consistency of skills taught to medical students and competencies achieved [21]. There was also evidence of a mismatch between skills taught and those necessarily required for practice [22]. Furthermore, students expressed dissatisfaction with their training [23].

Over the past twenty years, several studies have confirmed the inadequacy of clerkships to learn skills. Remmen et al. for example showed that students following a curriculum with an elaborate skills training programme in the pre-clinical phase practised significantly more basic clinical skills during clerkships [24]. Further studies showed that student performance of physical examination skills was unsatisfactory in curricula which relied on clerkships as the main teaching methods for those skills [25]. Longitudinal skills laboratory training and assessment in the preclinical years act as facilitators for skills training during clerkships later in the curriculum [26].

Furthermore, at individual student level learning experiences in clerkships differ substantially due to variations in patient mix, supervision and student numbers [27]. Studies of didactic processes during clerkships show that teaching of physical examination skills is often substandard [26,28-30].

In light of these findings and the lack of understanding how skills are acquired during the clinical phase of medical education we aimed to provide more insight into the process of learning physical examination skills in a work-based setting. We therefore pose the following research questions, to be addressed through qualitative methodology;

How do students describe the process of learning clinical skills in the workplace?

Which factors influence this process of learning?

## Methods

### Context of the study

This study was carried out at the Faculty of Health, Medicine and Life Sciences at Maastricht University, the Netherlands. The six-year medical curriculum consists of problem-based learning in the 3-year pre-clinical phase, followed by two years of clinical rotations in various departments. The final year consists of an 18-week research internship and an 18-week clinical internship in a single department.

Clinical skill training in the pre-clinical years is delivered in parallel with the six to ten-week block content. The skills training programme is developed, organised and delivered by the Skills Lab, a specialised educational facility. A training session typically lasts ninety minutes and is conducted in groups of eight to ten students. Students can prepare by studying recommended reading before training sessions. A typical training session consists of a four-stage process comprising demonstration of the skill by the teacher, explanation of the skill by the teacher, supervised practice (first on models/manikins, then on peers) and corrective critique [31]. During the clinical rotations students receive skills training that intend to refresh and rehearse the essential clinical skills that have been addressed in the previous years. At the end of year 3 there is a compulsory Objective Structured Clinical Examination (OSCE). Performance is scored on checklists by trained observers. Passing the OSCE is prerequisite for advancing to the next stage of the curriculum.

### Study design

We chose a qualitative approach of using focus groups to answer our research questions, as it allows for in-depth exploration of students’ experiences and it can include a wide variety of perspectives. We chose focus group discussions over individual interviews because we expected the interactions and group dynamics to provide richer data [32]. We organized the sessions according to the guidelines described by Morgan [33]. We used an interview scheme based on review of the literature. The interview guide was subsequently discussed within the research team and revised accordingly. It contained general open questions and prompting cues to be asked when elaboration of a topic did not occur spontaneously.

### Context

We chose to use the internal medicine rotation as the focus of our study, as it covers a wide range of clinical settings in which students participate. We expected that students would encounter a wide variety of patient problems and physical examination skills. No other hospital-based rotation delivers as much diversity as Internal Medicine, with both hospitalized and ambulatory patients who have undifferentiated or multi-system diseases.

At Maastricht University the Internal Medicine clerkship is organized in the fourth year and lasts ten weeks in total. The actual placement is preceded by a one-week introduction course where students receive a refresher on pathophysiology and commonly encountered complaints in internal medicine. During their time in the hospital, students will rotate through different services for 1–2 week each, e.g. out-patient clinic, ward, consultations, and in different sub-specialties where available, e.g. Pulmonology, Cardiology, Oncology. It is important to note that Internists are considered secondary care physicians in the Netherlands, and patients require a referral from their general practitioner.

Students will spend their days “shadowing” residents, and are expected to gradually take on more responsibility for patient care as they progress.

### Participants

As we did not want to interview students while they were still in the ‘shock of practice’; we deliberately invited students who started their rotations at least 6 months prior to the focus group sessions.

We purposively sampled our study participants by inviting students who completed their rotation in internal medicine. As the rotations in Maastricht University are in random order, every month a new batch of students enrols in the internal medicine rotation. We approached these students by email to participate later. All students received information about the study by RD through a presentation held at the final day of their rotation. All interviewees received written information about the goals of the study and the expected procedures. All participants signed a consent form. We provided lunch during the focus groups and students received remuneration for participation (25 euros).

In total, 32 students who had recently finished their internal medicine rotation participated in six focus group sessions (number of students per focus group ranged 4–9). At that point they had completed a median of 3 rotations. Of the participants 70% was female and 30% was male which is comparable to the overall male: female ratio at Maastricht University. The median age of our interviewees (22 years old) corresponds with the expected age of fifth-year medical students in the Netherlands. The test results of students participating in this study did not significantly differ from the averages scores in their cohort.

### Focus group discussions

All focus groups discussions were facilitated by one researcher (RS), who had no prior relationship with the students and was identified as assistant professor of medical education. A second researcher (RD) observed and took research notes, interrupting to ask additional questions if necessary. In total, we held six focus group sessions between February and May 2011. Each session was 80–90 minutes in length.

### Analysis

We started the analysis concurrently with data collection to ensure the elicited responses were in accordance with the research questions. Two researchers (RD and RS) debriefed after each focus group and discussed the principle themes that were mentioned in that particular session and contrasted these with previous sessions. This iterative process allowed us to estimate the point of saturation. Saturation was reached after five sessions; we held one more focus group to confirm this. With the consent of participants, we recorded the focus group discussions and transcribed them verbatim. RD made summaries based on the verbatim and the research notes taken during the focus group sessions. Participants were sent the summary and invited to review and make additions (member check). Only three students made use of this opportunity which resulted in minor adjustments to wording in our analysis. Three researchers (RD, JvD, RS) iteratively read and coded the transcripts individually and independently. The emerging core themes were compared and any discrepancies were resolved by consensus. One researcher (RD) identified, coded and analysed the themes and subthemes identified by JvD and RS using AtlasTi 6.2 software. A second researcher (RS) repeated the coding to enhance scientific rigour. The coding frame that developed was grounded in the data rather than decided a priori. The research team reviewed this coding frame and refined codes and categories through a process of constant comparison and axial coding. Codes were selected to illustrate the themes raised by participants and chose examples indicative both of typical responses and of the diversity of views obtained. We identified and discussed divergent examples within each theme. Identified subthemes during the final stages of analysis were considered in relation to relevant literature, for example work based learning [34]. Through this process of constant comparison we developed our emerging construct to answer the research questions.

### Ethical approval

At the time of study, educational research studies reporting students’ experience did not require approval from the ethics committee at Maastricht University. This study does not fall under the appropriate legislation concerning research on human subjects. However, relevant ethical issues were carefully considered by the Department of Educational Research and Development. All participants received written information about the purpose and procedure of the study, and were recruited on voluntary basis. Students were informed they could leave the focus group session at any time. There were no adverse consequences for not participating. We assured students that research data could not be traced to individual students, and that analyses would be used for scientific purposes only. All students gave informed consent and we have their signed agreement forms on file.

## Results

We categorized the findings from the initial analysis of the data under related themes. In the following paragraphs, we will clarify the learning process as described by students in our focus group discussions. We will discuss our findings by answering the following questions,

How do students describe the process of learning clinical skills in the workplace?

What is learnt?

How is it learnt?

Which factors influence this?

What is learnt?

### Systematics: how and when to use physical examination skills

Students described what is learnt by discussing the systematics of how and when to use physical examination skills.

They mentioned that the way they use their skills changes when they reach the clinical stage of their curriculum. Students reported to be more attentive to the purpose behind the physical examination and would tailor their thoroughness according to the context, e.g. a full physical in emergency department or a condensed version for pre-operative check. This approach is in contrast to earlier years, where doing a physical examination merely involved doing the right things in the right order according to the guidelines provided by the Skills Lab. They said to use the steps learned in Years 1–3 as stepping-stones in their patient encounters.

*“Yes, that’s right *[in the skills lab] *you put your stethoscope on those five spots. And then you think ‘I got that right!’. But now* [in the hospital] *you really think: ‘okay, that’s one heart sound, that’s the second. Is there a murmur? Where is it? Is it systolic?”* FG1P1

As it was not always clear what needed to be included in the physical examination and expectations varied amongst supervisors, some said the differences in required rigor caused confusion. Also, students reported to learn how to approach physicals in a systematic way so that a) they would not forget any step in the examination and b) they would not forget any findings. The expectations of the supervisor in reporting back findings are guiding student actions.

*“Reporting back, I think, to the doctors. If you have seen a patient yourself and there is no supervision, then you report back and you give a brief summary of what you have seen. What you think is going on, the diagnosis and so on. I also think that helps a lot, because you get questions when it is not complete. Was the CVP increased? Oops, I didn’t measure that”* FG3P4

### Agility

The agility with which students said to perform a physical examination increased, especially for difficult or intimate examinations. Depending on the clinical setting they were posted, students learned different aspects of the examination. Students made clear distinctions between the consequences of intimate examinations in the various hospital settings e.g. ward versus emergency department (ED). They realized that in ED a thorough physical is more important than on the ward. Students mentioned that patients on the wards were already examined before and there was little to be gained from yet another intimate exam.

“*The examination is driven more by your expectations – often you do it less intensely* [when not in ED] *because you wouldn’t expect to find anything”* FG4P1

Non-invasive examinations such as auscultation of the lungs or heart were examples of types of skills that students mentioned to practise during their ward duty.

### Recognition of pathological signs

A key learning outcome of their clinical rotations was the ability to recognize pathological signs. Students indicated to learn how to specifically be on the lookout for abnormal findings during physical examinations. They also described being increasingly more able to give meaning to these findings and how their ability to interpret them changed during their rotations.

*“And the patient with ileus, I had never heard abnormal bowel sounds so you don’t know what you’re looking for. So you listen and think: is this it? And then you hear it once and then you will know just what you are hearing. So the next time you are performing abdominal auscultation you think; ah, these are normal bowel sounds, peristaltic.”* FG3P2

### How is it learnt?

Students reported a wide variety of activities that contributed to their learning. Our analysis revealed a diverse range that upon closer inspection showed similarities to the work of Eraut who described ways of learning in the workplace [34]. We have derived the headings of our main themes from Eraut and adapted them to the workplace of the clinical internal medicine department; working alongside others, tackling challenging tasks and working with patients.

### Working alongside others

Interviewees described to learn by observation and developing your own way. They commented on the ways they learnt the correct procedures for physical examination. In relation to the preparatory training sessions held at the Skills Lab, students remarked that they experienced differences between the way they were trained to perform physical examination skills and the way they observed their supervisors act.

They mentioned that their adopted ‘own best strategy’ often resulted from the idiosyncrasies of their supervisors. That is, students felt they needed to know not so much the right way to do something but the way preferred by the supervisor. Students talked about the development of their own individual way of performing physical examination skills by comparing different examples and contrasting that with their own experience.

*“So every time I was shadowing a different doctor I thought, oh this one does it this way, that’s quite handy. So you pick up different techniques every time by watching them and on the ward is where you can really put it into practice and see how it is. I really learned the most there.”* FG4P1

Students mentioned that a strong motivator to execute the physical examination well was the prospect of having to make their findings explicit and report them back to their supervisor. They described to “force themselves” to do the exam carefully and with great attention – this was especially true when they received direct observation. Explicitly stating their findings – or doubts – enabled them to discuss the interpretation and to expand their understanding.

*“No, what I often did, was listening to the lungs together and then he* [the supervisor] *said well, describe what you are hearing. And that’s just very helpful.”* FG4P5

*“But also because you have to write in the patient file and make it explicit what you’ve heard, so to say. That made me pay attention during auscultation, and already thinking about what I would write down later.”* P1FG1

### Tackling challenging tasks

Students discussed the role of responsibility in their growing conscientiousness. The described change in students’ attention towards abnormal signs during physical examination is influenced by two considerations. On the one hand they do this because they think it is expected since they will have to report back to their supervisor either verbally or in writing in the patient file. On the other hand students look for abnormal signs during physicals because this allows them to play an important role in the diagnostic process of the patient they see.

*“After a few times examining patients under supervision, they all said; well I trust you, you can do that. And then you go to the next patient on your own and think: he is not going to verify my findings. Now I have to get it right.”* FG4P2

During their time in clinic, students learn from discussing abnormal findings. They build a personal archive of pathological findings that they extend in two ways; either by being active in looking for these signs in patients they see and assertively asking for feedback on their findings, or by being directed to them by their supervisors. This latter approach might mean both seeing a patient together and performing the physical examination with direct feedback, or being told by the supervisor to go and see a particular patient again on their own.

*“And that* [identifying gurgling abdominal sounds] *in a patient is far more impressive than hearing them on a CD, because that does not stick”.* FG2P4

Students also reported more specifically on the role of assertiveness in asking for feedback or supervision. When there is little encouragement from supervisors to discuss findings and to verify doubts, this may lead to uncertainty about the correctness of their execution of physical examination skills. Reasons for this include insufficient time or access to the supervisor. When confronted with such a situation, most students expressed hesitation to actively seek feedback due to lack of confidence to persist.

“[I would ask my attending:] *Would you mind repeating the auscultation to check? That works quite well, you gain more confidence this way when they confirm, yes, this really is a heart murmur. But you have to ask, sometimes really urge or even badger as it is not common to be observed”. FG3P1*

Students described how the nature of the tasks changed during their rotation. Some said to primarily see patients independently completing history and physical examination before seeking supervision while others reported spending much time on administrative tasks or simple procedures such as venapunctures. Taking responsibility often emerged through individual students’ initiative, although the participants also indicated that supervisor behavior was important as they provided them with the opportunity to engage in seeing patients at outpatient clinics or at ED and to undertake a full physical exam. This was seen to enhance both participation and learning. However this also brought the obligation of being clinically competent, or at least appearing to be. This seems to be a reinforcing cycle, whereby students with a proactive attitude are provided with more learning opportunities and increasing responsibility. Especially in departments with many students this may lead to a hierarchy of learners according to their level of proactivity, with students near the bottom needing experience but not getting it.

### Working with patients

Students mentioned that seeing patients and being able to link patients’ previous history and anamnesis with physical examination findings was a strong driver for learning. Through reflection on the findings, alone or with the help of a supervisor, students begin to construct an understanding of these clinical experiences. They begin to attach meaning to concepts they previously had no notion of or had difficulty comprehending. The opportunity to compare several patients in a relatively short time span helped them to develop the ability to distinguish between physiological (normal) and pathological findings.

*“Like how a normal abdomen sounds, for that you have to have listened to many bellies. Here in the skills lab you may have examined one or two of your fellow students’. But to really be able to distinguish between normal and abnormal, you just have to do it* [abdominal examination] *many times.”* FG2P3

### What factors influence this?

#### Factors associated with the physical examination itself

Students described mediating factors that helped them in their learning process of physical examination skills. One of the most frequently mentioned factors was the physical environment where learning takes place, e.g. outpatient clinic, ED or wards. Each setting has its own unique set of learning opportunities, but not all students were able to spend time in each because of how the clerkship is structured (see context in method secion). Students mentioned to use the differences in setting, patient population, delivery of care etc. to practise different parts of their physical examination skills. They emphasized the effect of the role they are assigned during their rotation as contributing to their learning. Being able to contribute to the diagnostic process by examining patients independently for the first time at the ED or outpatient clinic, provided strong incentives for learning.

*“Well, it’s often that there are many more things that you can detect at ED as compared to when a patient comes to the out-patient clinic. Then patients have already been seen by the GP or the patient is already known and comes for a follow-up visit. Then you have nothing more to find out, to discover, so to speak. While at the ED, you can find out much more by yourself and you’re the first one to think, what is this, what could it be and what can we do. That’s nice.”* FG4P2

*“It’s also because students see new patients and need to chart them. You can learn something from that. But with patients who have already been seen, then you are just repeating the examination. We’ve talked about this earlier, about repeating something that is already been done, but you learn more when you’re actually doing something new.”* FG5P3

Supervisors can play a mediating role in this by clearly defining the role of students in their department and making sure necessary organizational needs are met (e.g. dedicated supervisor, patients scheduled for student consultation, own room). They can also facilitate students learning by encouraging them to ‘tag along’ during morning rounds to help interpret pathological findings. Students found teaching behavior that stimulates their engagement motivating, and offered examples of ways to include students in clinical encounters. Simple actions such as allowing students to examine the patient first versus observing the supervisor gives them an opportunity to reflect on the findings and challenge their ability to describe them appropriately.

*“So if you have to really think about it or the answers are already laid out for you and you only need to go and check it, well yes indeed. That makes a lot of difference.”* FG4P1

The ability to recognize pathological findings depends on the opportunity to examine patients displaying these; this learning is largely serendipitous by nature. Often it is left to individual proactivity to look for interesting cases. However, students offered examples in which this type of learning was offered in a systematic and planned fashion, e.g. during morning rounds.

*“We really had to go and, hey I hear that a patient was admitted here with an ileus and then you’d better go there and take a look.”* FG1P5

### Related to individual learners’ characteristics

Our focus groups revealed that learning is hampered by uncertainty resulting from students seeking their place and position in the department. A wait-and-see attitude results from students being preoccupied with questions such as “am I doing things right?”. They kept themselves in the background, often to the advantage of those with a proactive attitude. In departments with many students, or sixth year students, this leads to lost learning opportunities when a ‘queue’ of learners forms with the more senior or proactive monopolizing interesting tasks.

Factors influencing students’ confidence in detecting abnormalities during physical examination are already mentioned above, namely the opportunity to build a personal frame of reference or archive and direct feedback when there is uncertainty. In addition to those factors, our interviewees identified another facilitator to their learning. In the focus groups they called it ‘proactivity’, ‘assertivity’ or ‘being forward’. Irrespective of the terminology they all refer to the notion of being able to influence the outcome of events by one's individual actions. In other words, students who are able to speak up, offer to undertake tasks and actively seek participation are more likely to see a wide variety of patient cases. They will have had more opportunity to practise the execution of the physical examination and they will have seen a larger number and wider variety of abnormal findings. Moreover, student reported that supervisors often respond more favourably to ‘outspoken’ students (extravert, assertive, enthusiastic) and provide them more easily with learning opportunities.

“*When you heard for example that there was a patient with an interesting diagnosis or abnormal findings on physical examination, I would just go there, chat with them and ask whether I could do the examination again so you can learn from it.”* FG6P2

*“When I was charting patients and I was like, oh this might be an abnormal heart sound, you know I have a limited frame of reference so there was always someone who came along and would later explain it to me. But often you had to know who to ask and pick the right person for that.”* FG2P4

*“Would you just listen if it is correct? That worked most of the time, then you would get some more experience. That makes you more confident, like, that you just know okay this really is a murmur. But the thing is you really have to ask for someone, it’s not that if you are actually going to be observed.”* FG1P5

### Contextual factors

The contextual factors that influence learning of physical examination skills relate to overcoming the hierarchy of learners and by promoting proactivity. Incorporating the factors discussed earlier, contextual influences on student learning concern the physical context and social context of the clinical rotation.

The physical context refers to the physical setting in which learning takes place, and we have already discussed the differences between the outpatient clinic, ED and the wards. Students mentioned that all three settings afforded different learning experiences. As mentioned earlier, seeing new patients at the ED enabled students to work independently and with an actual contribution to patient care whereas seeing patients in the outpatient clinic was conducive for learning only if there was ample opportunity for feedback.

*“I participated in the teaching outpatient clinic and when I saw a patient I was given my own room where I could work until I was finished and then I asked the consultant to come and have a look. But I could just as easily take my time and spend half an hour taking the history if I thought that was necessary. So I was given space and time there to do my own thing.”* FG5P2

On the ward, the focus of learning shifted from diagnostic skills to patient management skills which – although important – fall outside the scope of this study. The accessibility of patients on the wards gives students plenty opportunity to compare different variations of physiology and pathology. While any of these types of learning can occur in any setting, students recognized that some contexts were more likely to provide opportunities than others. For example, some students explained the lay-out/arrangement at their hospital which had a designated room at the outpatient clinic for student use, where they could see patients on their own with ample time to do so and the possibility to discuss their findings with an approachable supervisor.

*“Yes, that makes a real difference. With some doctors it’s really nice, they count on you to be there and you get to see patients on your own and do everything yourself and discuss it. With others, well, you’re just there, listening in.”* FG1P1

The social context refers to the ways the people involved in the rotation shape learning, attendings, residents, fellow students, and nurses. We have already pointed out some aspects of the social context, such as the hierarchy of learners and the role students are assigned. Other influences include the effect of supervision and the proactivity discussed earlier.

Teachers can directly affect the learning outcomes by role-modelling or providing direct feedback. Also, the explicit invitation to participate in the work is an important aspect of the influence teachers can have. Students said they needed to feel valued and be allowed to approach their supervisors; if these conditions are not met students may not participate fully and lose learning opportunities.

*“It has to come from both sides. An assertive attitude is important, that will keep you busy for a while. But if it’s all up to you, then at some point you will think, okay now I have done enough, I’ve had it, just leave it.”* FG3P2

Students reported a wide variety between the amount of introduction and guidance in their respective departments. Some said to have had no schedule and every day was open to do whatever they felt like, while others stated to have fixed rotations within the department while being assigned to a particular member of staff. Overall, most students favoured the structured context as it provides direction and stimulus. Some expressed the desire for more responsibility within their range of existing competence and development, as they sometimes felt under stimulated.

*“Because it is quite easy to do very little. When you are not all that assertive by nature and you ask for it, you end up doing very little and some residents really pick up on that and just say: you can do this or you can go do that.”* FG2P1

## Discussion and conclusions

### Relationship of principal findings with literature

This study set out to explore the learning process of physical examination skills during clinical rotations. We have identified the nature of the learning outcomes and investigated how the characteristics of the individual student and the physical and social context in which learning occurs influence this process. Students report to learn the systematics of the physical examination, gain agility and become able to recognise pathological signs. The learning process involves a combination of working alongside others and working independently with increasing responsibility for patient care.

Our results underline the social nature of learning physical examination skills; no supervisor no matter how good can create a good learning environment without input from the learner. No student no matter how proactive can force learning to happen without the prerequisite support from the supervisor and the environment. This forefronts the concept of relational interdependence as described by Billett [35] where individual agency and social aspects of the learning environment collide. Besides the fact that supervisors have the power to afford (i.e. have access to) learning experiences, the individual learner needs to navigate these affordances and align them with his/her personal learning needs. Moreover, participants in our study stressed the ‘social genesis’ of learning in the workplace in their description of learning physical examination skills by mimicking the behaviour of supervisors in order to demonstrate that they belong in the environment and merit a favourable assessment.

Furthermore, the observations made by our participants are in accordance with the notions of situativity theory [36]. Its key tenet is that knowledge and thinking as well as learning are situated in experience. It stresses the social nature of learning, with emphasis on the importance of the participants and their environment as well as the evolving interaction between them.

### Inter-student variation of experiences

Based on our focus group discussions there seems to be huge inter-student variation when it comes to clerkship experiences, an observation confirmed by other studies [16,37-39]. The characteristics of the student are a determining factor of the actual learning that takes place; this is especially true for how often they receive observed and unobserved feedback. This complex interaction between students and their environment means that the notion of ‘curriculum’ in the clinical context is a purely hypothetical one. A characteristic of workplace learning is that the education actually being offered (curriculum in action) might differ from the formal educational programme (curriculum on paper). What students actually learn during their clinical rotations (experienced curriculum) is different again and will even differ between two individuals who share the same experiences [40].

### Proactivity

Proactivity emerged as a key factor influencing individual students’ learning experiences. Our students reported difficulty in steering their own participation in the clinical workplace. Billet acknowledges the active role learners play in choosing how they participate and in what activities they participate [41]. To understand the personal relations between the learners and the social environment we need to highlight the work of Bandura, whose social cognitive theory not only is consistent with the situativity perspective but also explains students’ proactivity [42]. Central in his work is the concept of ‘personal agency’; the belief that individuals can influence the outcomes of events especially if other people are involved. Therefore when engaged in tasks individuals ‘cannot simply sit back and wait for the appropriate performances to appear’ [43], they need to regulate their thoughts and actions to benefit from the ongoing events.

In order for students to be able to be proactive, and seek out learning opportunities that will help them to obtain physical examination skills, they will need to develop self-regulatory behaviour. A central premise however, is that the students need to believe they can perform well. This belief is called perceived self-efficacy, and according to Bandura’s theory people with high self-efficacy are more likely to view difficult tasks as something to be mastered than something to be avoided. Research in medical education has shown however that students display poor capacity for self-assessment [44,45], and the transition into the clinical phase further reinforces students’ feelings of inadequacy [46].

On the specific topic of physical examination skills, Mavis has reported that students with high self-efficacy were more likely to score above the mean OSCE performance compared to low self-rated students, however there was no significant correlation [47].

### Participation in learning

It could be tempting to conclude that the problem (and hence the solution) of being proactive about learning physical examination skills lies solely with the individual student. That is a fallacy. As we have shown, the tangible outcome of notions such as proactivity and self-efficacy is student participation in the workplace. It can therefore not be the sole responsibility of the student to make this happen.

There is much emphasis in the field of medical education on the central role of participation in learning. A model of experience-based learning shows that ‘supported participation’ is essential in clinical workplace learning [15]. One of the defining characteristics of ineffective workplace learning is lack of participation. This is where a learner seems passive, unwilling to learn or just does what he or she is told [48]. Our findings suggest that once students are assimilated in the clinical department and have a supported role in the day-to-day functioning of the workplace they are more eager to engage. This is facilitated by having overcome the ‘shock-of-practice’; the participants with more clinical experience (more completed rotations) stressed how some awareness of the expected code of conduct makes it easier to participate in the clinic [49].

Dornan concludes that student participation is shaped by department-related factors and by students’ “human interactions”. Our results show similarities to his model, most notably when students elaborated on the student-supervisor relationship. Our findings suggest that observation of physical examination skills during clinical rotations is scarce. Most of the time supervision is not based on direct observation but apparently inferred from vicarious information. Examples that were mentioned in our focus groups include the oral reporting of findings, or the written records in patient files. Previous studies have also suggested that supervision is a rather haphazard learning event [50-52].

From our findings and their relationship with the literature we can derive implications for current practice.

### Implications for current practice

The contribution of this study is firstly conceptual in nature by describing the learning process of physical examination skills during clinical rotations. It provides a better understanding of the complex nature of learning in workplaces and as such expands the existing literature on this topic in the field of medical education. Secondly, the results of our study allow us to discuss and analyse the quality of learning experiences during clinical rotations. Our contributions can be used to improve the quality of existing programmes and to guide the design and implementation of new ways to learn physical examination skills.

The implications of this study for current practice are threefold; on the level of the individual student, the level of hospital department and the level of university.

On the level of the individual student, our findings revolve around the interaction between the individual student and his environment in which participation is pivotal for learning. Educational interventions should encourage students to adopt a proactive attitude, take initiative to enhance their own learning and be responsible for their own performance. Possible examples include pre-rotation workshops in self-regulatory behaviour and proactivity. This strategy could be strengthened on the level of the department hosting the clinical rotation, by making practice more accessible to newcomers. Initially, students can be introduced into the clinical workplace by allowing them to observe (e.g. in an outpatient clinic), followed by allowing them to perform low-risk tasks (e.g. physical examination on the ward) or by letting them perform under close supervision (e.g. ED). Such a process highlights the importance of not simply assuming the level of competence based on years of training, but advocates for a learner-centred approach based on the actual level of competence a student has gained.

Our findings suggest that although the principles of supervision according to this model are somewhat present in clinical practice, they do not meet individual students’ needs. Special attention needs to be given to ensure that students carry clinical responsibility according to one’s means, without compromising patient safety.

One of the first steps to promote such a positive learning environment is to provide an effective initiation or orientation; this includes practical information, clarification of the roles of students and a clear description of the expectations that others have in relation to their performance [53].

Good clerkship organization can be bolstered at the level of the university or course directors. Support can include faculty development courses [54], and the provision of the preparatory workshops for students entering the clerkships [55]. Special consideration can be given to expectation management (both of clinical staff and students) [56] and alignment of educational goals to be achieved in the rotations [57].

Other issues that need to be centrally organized to deliver a superior clinical programme, especially when using satellite hospitals or rural placements, include comprehensive quality assurance and feedback on performance of departments and individual supervisors [58].

### Limitations of the study

This study explored student experiences in a select population, namely one medical school. We acknowledge the limitations this design may have on the outcomes of the study. Our training programme for clinical skills in the preclinical phase is more integrated and extensive than that found in other schools [59,60].

It has been demonstrated that our students enter the clinical phase of their curriculum with a better preparation and higher level of skills mastery than in school without, or with a different skills lab [61]. Although we recognize the effect this has on the generalizability of our findings, we accentuate that the actual deficiencies in clinical learning experiences may be underestimated as a result.

Secondly, we rely on self-reports to describe the learning process of physical examination skills. These are not gauged against any objective measurement of actual performance in practice. We are aware of the lack of external validity our approach causes. We have deliberately chosen to analyse the underlying process instead of focusing on outcomes, but we urge future research efforts to investigate this relation. Nevertheless, we think self-reporting is a strength too, since the impact of various influences on practising behaviour will be mediated through self-perceptions.

Thirdly, the sample size of our focus group studies is small. The presence of theoretical saturation, coupled with the diverse range of reactions from our interviewees suggests that we have assured representativeness. We are aware that qualitative research brings about the issue of observer dependency during data collection, and confirmation bias during data analysis. By adopting the iterative process described in the Methods section we guaranteed scientific robustness.

### Conclusions and future research

This study explores learning skills in clinical rotations; a previously neglected area of research in the field of workplace learning. Our findings suggest that although individual student experiences vary greatly between different hospitals, it seems that proactivity and participation are central drivers for learning. This study adds to a growing body of literature on the process of learning during the clinical phase of medical education. Our results are especially relevant in light of existing interventions and current innovations aimed at improving the quality of clinical rotations [62]. Future research should focus on the actual performance of skills, as well as the role of preparation in pre-clinical years on the desired learning outcomes.

## Competing interests

All authors declare that they have no competing interests. RD has received a Kootstra Talent Fellowship to pursue PhD research, but the granting body had no role in the design of this study nor the preparation of this manuscript before submission.

## Authors’ contributions

RD, RS and JvD designed the study, with contributions from AS and CvdV. RD and RS carried out data collection and RD, RS and JvD analysed the results. All authors discussed the results. RD wrote the first draft of the paper, with extensive feedback from RS. All authors provided comments and revisions and approved the final manuscript.

## Pre-publication history

The pre-publication history for this paper can be accessed here:

http://www.biomedcentral.com/1472-6920/14/61/prepub
